# Editorial: Developing Lifestyle Entrepreneurship for Sustainable Destinations

**DOI:** 10.3389/fpsyg.2022.970005

**Published:** 2022-07-13

**Authors:** Alvaro Dias, Biagio Simonetti, Fiona Eva Bakas

**Affiliations:** ^1^Universidade Lusófona and ISCTE-IUL, Lisbon, Portugal; ^2^University of Sannio, Benevento, Italy; ^3^Universidade Lusófona, Lisbon, Portugal

**Keywords:** sustainability, SMTEs, innovation, competitiveness, destination competitive advantage

This special issue aims to promote the discussion around lifestyle entrepreneurship which, although being important, as presented in the commentary by Dias and Patuleia, little attention has been paid to the subject. In specific, it is intended to know this specific group of entrepreneurs in more detail in its dimensions of attraction factors, innovation and business development associated with lifestyle.

The impact has exceeded expectations. Considering May 2022 data, in 1 year, the special issue had 1,591 article downloads and 12,072 article views, with the total views increasing six times in that period, from 2,500 to 15,000.

The first study is devoted to poverty alleviation in ethnic regions prepared by Yang et al. analyses eight ethnic provinces in China and combines the Data Envelopment Analysis model with the Malmquist index to assess the impacts of nascent tourism investment on poverty alleviation. The authors explore these impacts on the two stages of tourism poverty alleviation. The findings of the paper reveal that tourism has a positive impact on the local economy and on improving the living standards of local people. However, the efficiency of the measures needs to be refined and the authors present various ways to achieve this.

Linderová et al. present a study on local people's attitudes toward tourism development, with an empirical study carried out in eastern Czechia, more specifically in the urban monument zone of Predhradí. The authors drew on survey data collected from residents aiming to assess the positive and negative impacts arising from tourism development. Despite the problems identified such as pollution of public spaces and inappropriate behavior of tourists, residents recognize that this is part of the progress, benefiting from job creation and heritage preservation, highlighting also the implications on local entrepreneurship.

The practices of community-based tourism (CBT) in elephant habitat communities were studied by Lo and Janta in northern Thailand. The authors recognize the role that this type of tourism plays in terms of sustainable development, noting that it allows the negative aspects of tourism (waste, unemployment) to be overcome through the participatory management of natural resources and the involvement of the community and public entities.

This special issue also includes a commentary on the second study, highlighting the role of the supply side, as the study by Linderová et al. focuses on the resident side. In this commentary, Dias and Patuleia highlight the role of the entrepreneur in community involvement, and preservation of the local way of life and environment, pointing out the need to stimulate small businesses and recognize the limitations that local entrepreneurs may have.

The theme of lifestyle entrepreneurs presents a wide range of possibilities for future research as observed in the various studies in this special issue. Of particular interest is the attention given to community development, whether in poverty alleviation, CBT, or the involvement of a community in the development of a tourism project. Indeed, the embeddedness of the entrepreneur in the local context is determinant to offering innovative experiences (Yachin, 2019), accessing local knowledge, source of new opportunities for product development and narratives (Dias et al., 2021b) and developing the network with other small businesses whose complexity makes the tourism product unique (Richards, 2011).

It was also noted the importance that lifestyle entrepreneurship has in the innovation of tourism destinations, not only as a result of its local embeddedness (Bredvold and Skålén, 2016) but also by the very essence of the concept, where the demand for a particular lifestyle can contribute to differentiation in the experiences offered and a driver of the population's wellbeing as unanimously verified in all articles. An interesting aspect highlighted in all articles is the role of public entities in the process of local entrepreneurship development, which is in line with previous studies (Dias et al., 2021a).

This special issue certainly does not close the subject, on the contrary, it points out paths for future research, especially for the perception of the phenomenon of lifestyle entrepreneurship through the intersection with other themes such as alleviating poverty, creativity, CBT or even responding to the challenges for greater sustainability in a post-pandemic context such as degrowth (Higgins-Desbiolles et al., 2019).

## Author contributions

All authors listed have made a substantial, direct, and intellectual contribution to the work and approved it for publication.

## Conflict of interest

The authors declare that the research was conducted in the absence of any commercial or financial relationships that could be construed as a potential conflict of interest.

## Publisher's note

All claims expressed in this article are solely those of the authors and do not necessarily represent those of their affiliated organizations, or those of the publisher, the editors and the reviewers. Any product that may be evaluated in this article, or claim that may be made by its manufacturer, is not guaranteed or endorsed by the publisher.
